# Effect of ectoparasite *Ichthyophthirius multifiliis* on the histopathology and gill and gut microbiota of goldfish (*Carassius auratus*)

**DOI:** 10.3389/fvets.2025.1539446

**Published:** 2025-02-04

**Authors:** Xialian Bu, Xianqi Peng, Lei Huang, Yu Zhao, Jinbiao Jiao, Jian Zhu, Jing Chen, Xiaohong Huang, Aqin Zheng, Huantao Qu, Jiayun Yao

**Affiliations:** ^1^Hubei Key Laboratory of Three Gorges Project for Conservation of Fishes, Chinese Sturgeon Research Institute, China Three Gorges Corporation, Yichang, China; ^2^Key Laboratory of Healthy Freshwater Aquaculture, Ministry of Agriculture and Rural Affairs, Key Laboratory of Fish Health and Nutrition of Zhejiang Province, Key Laboratory of Fishery Environment and Aquatic Product Quality and Safety of Huzhou City, Zhejiang Institute of Freshwater Fisheries, Huzhou, China

**Keywords:** *Ichthyophthirius multifiliis*, microbiota, histopathology, opportunistic pathogens, ectoparasite

## Abstract

**Introduction:**

The ectoparasite *Ichthyophthirius multifiliis*, is the pathogen of white spot disease in freshwater fish, which parasitizes on gills, fins, and skins of fish, causing tissue damage and death of host. However, whether it influences gill and gut microbiota is still unknow.

**Methods:**

In this study, H&E staining was used to show the gill and gut histopathological characteristics of *I. multifiliis*-infected and uninfected goldfish (*Carassius auratus*). Meanwhile, 16S rRNA gene amplicon sequencing was conducted to analyze the difference of gill and gut microbiota between *I. multifiliis*-infected and uninfected goldfish.

**Results:**

Histopathological examination revealed that *I. multifiliis* has induced significant damage to the gills of goldfish, characterized by lamellae fusion, cell hyperplasia, cell hyperaemia, inflammatory infiltration, necrosis and desquamation. 16S rRNA gene sequencing result showed that alpha and beta diversity of gill microbiota was significantly reduced in the *I. multifiliis*-infected group, while no significant changes were observed in gut microbiota. Genus *Candidatus Megaira* exhibited the highest relative abundance in the *I. multifiliis*-infected group. Meanwhile, the abundance of opportunistic pathogens *Aeromonas* and *Achromobacter* were increased in the intestines of *I. multifiliis*-infected goldfish.

**Discussion:**

The increased presence of *Candidatus Megaira* may originate from within the cells of *I. multifiliis*. The increase of opportunistic pathogens *Aeromonas* and *Achromobacter* may pose a threat to the health of goldfish. In summary, this study laid a foundation for further research on the interaction between *I. multifiliis* and host microbiota.

## 1 Introduction

Ichthyophthiriasis is one of the most severe fish diseases of both wild and cultured freshwater fish, which results in significant economic losses in global aquaculture industry (1, 2). This disease also known as white spot disease and the pathogen of it is *Ichthyophthirius multifiliis*, a ciliated protozoan (3). Life cycle of *I. multifiliis* consists of four developmental stages including a parasitic trophont, a free-swimming protomont, a reproductive tomont, and an infective theront (4, 5). When it parasitizes fish’s gills or fins, it can easily invade fish epithelial cells and feed on the cells, mucus, and the tissue fragments, leading to severe tissue damage, increasing the opportunity of secondary infection, and causing a large number of host death in a short time (6, 7). However, there is still no safe and specific drugs for the prevention and control of Ichthyophthiriasis. It is urgent to find effective treatment methods to support the healthy culture of freshwater fish.

Microorganisms can be found on the fish’s skin surface, gills, fins, and in its gastrointestinal tract, which play important roles in host homeostasis and physiology (8). For instance, gut microbiota can produce short-chain fatty acids and contribute to hosts’ metabolism (9). It also shows fundamental roles in protection against pathogen invasion (10). However, apart from these beneficial commensal and symbiotic microorganisms, fish also face threats from pathogenic bacteria. Parasitic infection and exposure are likely to induce changes in fish’s microbiota. Research has shown that *Dactylogyrus lamellatus* infection can significantly reduce the diversity of the gut microbiota and increase the relative abundance of *Cetobacterium* in grass carp (11). An infection of endoparasite *Khawia japonensis* in common carp could lead to the increase of two pathogenic bacterial genera, *Lawsonia* and *Plesiomonas* (12). Protozoan *I. multifiliis* infection also led to a decreased abundance of skin commensals and increased colonization of opportunistic bacteria in rainbow trout (*Oncorhynchus mykiss*) (13). However, little is known about the interaction between gill and gut microbiota, and ectoparasite *I. multifiliis*.

Goldfish (*Carassius auratus*), is a popular ornamental species from all over the world. However, it is highly susceptible to *I. multifiliis* infection. This parasite can form visible white spots on fish gills, fins, and skins, affecting the ornamental value of goldfish and causing significant economic losses (14). Thus, goldfish is an ideal model organism for *I. multifiliis* infection.

In the present study, based on the goldfish infection model, the relationship between gill and gut microbiota, and *I. multifiliis* was characterized by 16S rRNA gene amplicon sequencing. The histopathological changes of gill and gut were observed and measured with the scoring system (15). These results demonstrated the relationship between gill and gut microbiota and *I. multifiliis*, which are beneficial for prevention and control of *I. multifiliis* infection in aquaculture.

## 2 Materials and methods

### 2.1 Parasite infection and parasite burden quantification

Goldfish weighting 15–18 g, were purchased from a commercial supplier in Huzhou City, Zhejiang Province, China. The goldfish were kept in a large aquarium with a water temperature of 25 ± 2°C. To remove all ectoparasites and ensure no parasitic infections, fish were treated with three consecutive baths in 1:10000 formalin solution for 12 h at 48-h intervals. Then fish were cultured in 100 L aquariums with a water temperature of 25 ± 2°C. They were fed once a day with commercial fish pellet feed at 1% of their body weight.

Heavily infected goldfish from the laboratory were selected for trophonts collection. Then place the goldfish in a 1 L transparent box to allow trophonts to shed naturally. Subsequently, use a glass micropipette to collect the trophonts and place them into the tanks containing 90 healthy goldfish (30 goldfish per tank). Use a counter to tally the number of trophonts, with a final count of 500 for each tank. Another 30 healthy goldfish were set as control group. Both infection group and control group were kept under the same conditions as described above. The infection experiment lasted for 2 weeks. Monitor the behavior of the goldfish at 24 h intervals and randomly select 3 goldfish for microscopic examination to count the number of trophonts on their caudal fins.

### 2.2 Tissue sampling

Samples were collected on the fourteenth day of the experiment. All the experimental procedures and animal care were performed according to the protocols approved by the Institutional Animal Care and Use Committee of the Zhejiang Institute of Freshwater Fisheries. First, fish were anaesthetized with 0.02% tricaine methane sulfonate (MS-222, Sigma) according to manufacturer’s instructions. Then gills and hindgut content were isolated and collected under sterile conditions, then were frozen in liquid nitrogen and stored at −80°C for 16S rRNA gene amplicon sequencing.

### 2.3 Hematoxylin-eosin staining

Hematoxylin-eosin (H&E) staining was performed to assess the damage caused by *I. multifiliis* to the host gill tissues. First, gills were fixed in 10% neutral buffered formalin. After 48 h of fixation, gills were sectioned into cassettes, dehydrated through a series of graded ethanol solutions, cleared in xylene, embedded in paraffin wax and sliced into 5 μm thick sections and stained with HE. Then HE stained slides were examined using Axioplan 2 imaging and Axiophot 2 (Zeiss, Oberkochen, Germany). Histological changes like lamellae fusion, cell hyperplasia, necrosis, hyperemia, and desquamation were assessed according to the scoring system proposed by Baums et al. (15). Briefly, the score ranging from 1 to 7, represents the degree of change (1 - unchanged, 3 - mild, 5 - moderate, 7 - severe).

### 2.4 DNA extraction and 16S rRNA gene amplicon sequencing

Extraction of microbial DNA from gills and hindgut content samples was performed using the QIAamp DNA Stool Mini Kit (Qiagen, Germany) according to the manufacturer’s instructions. The DNA was detected by 1% agarose gel electrophoresis and a NanoDrop^®^ ND-2000 spectrophotometer (Thermo Scientific Inc., United States). Then hypervariable region V3-V4 of the bacterial 16S rRNA gene was amplified with primer pairs 338F and 806R (The forward primer 338F: 5′-ACTCCTACGGGAGGCAGCAG-3′, the reverse primer 806R: 5′-GGACTACHVGGGTWTCTAAT-3′) using an ABI GeneAmp^®^ 9700 PCR thermocycler (ABI, CA, United States). The PCR mixture was including 10 μL 2 × Pro Taq, 0.8 μL each primer (5 μM), 10 ng/μL of template DNA, and ddH_2_O to a final volume of 20 μL. The amplification conditions were as follows: initial denaturation at 95°C for 3 min; 25 cycles of denaturing at 95°C for 30 s, annealing at 53°C for 30 s and extension at 72°C for 45 s; and final extension at 72°C for 10 min. The PCR products were extracted from 2% agarose gel, purified using the AxyPrep DNA Gel Extraction Kit (Axygen Biosciences, Union City, CA, United States) and quantified using the QuantiFluorTM-ST Blue Fluorescence System (Promega, Beijing, China). Library was constructed by TruSeq^™^ DNA Sample Prep Kit (Illumina, California, United States) and subjected to sequence on an Illumina NextSeq 2000 platform to generate 300 paired-end reads.

### 2.5 Analysis of 16S rRNA gene sequences

The raw data were analyzed through the free online platform of majorbio cloud platform (cloud.majorbio.com). QIIME 2 V.2022.2 (16) was used to conduct Amplicon Sequence Variants (ASVs)-based analysis. First, reads were quality-filtered and denoised using qiime DADA2 denoising plugin to obtain the ASVs. Then ASVs were assigned taxonomic labels using qiime classify-sk-learn and SILVA database V.138 (17).

Alpha diversity was assessed using metrics including richness, shannon, phylogenetic diversity and pielou evenness indices. All the indices were calculated using Mothur V.1.30.2 (18). Beta-diversity was measured using Bray-Curtis distance. Principal Co-ordinates Analysis (PCoA) was carried out based on the beta-diversity index matrix to study the differences in sample community composition. Taxa were compared between *I. multifiliis*-infected group and control group by Linear discriminant analysis Effect Size (LEfSe). For the function prediction, KEGG annotation was conducted with the help of PICRUSt2 V.2.2.0 (19).

### 2.6 Statistical analysis

Kruskal-Wallis test was used to evaluate the intergroup difference of alpha diversity. Pairwised Wilcoxon Rank Sum test was used to determine microbiota that significantly differed between *I. multifiliis*-infected group and control group. All the statistical analysis were conducted in R language (version 3.3.1) (20).

## 3 Results

### 3.1 Infection status

**Figure 1:**
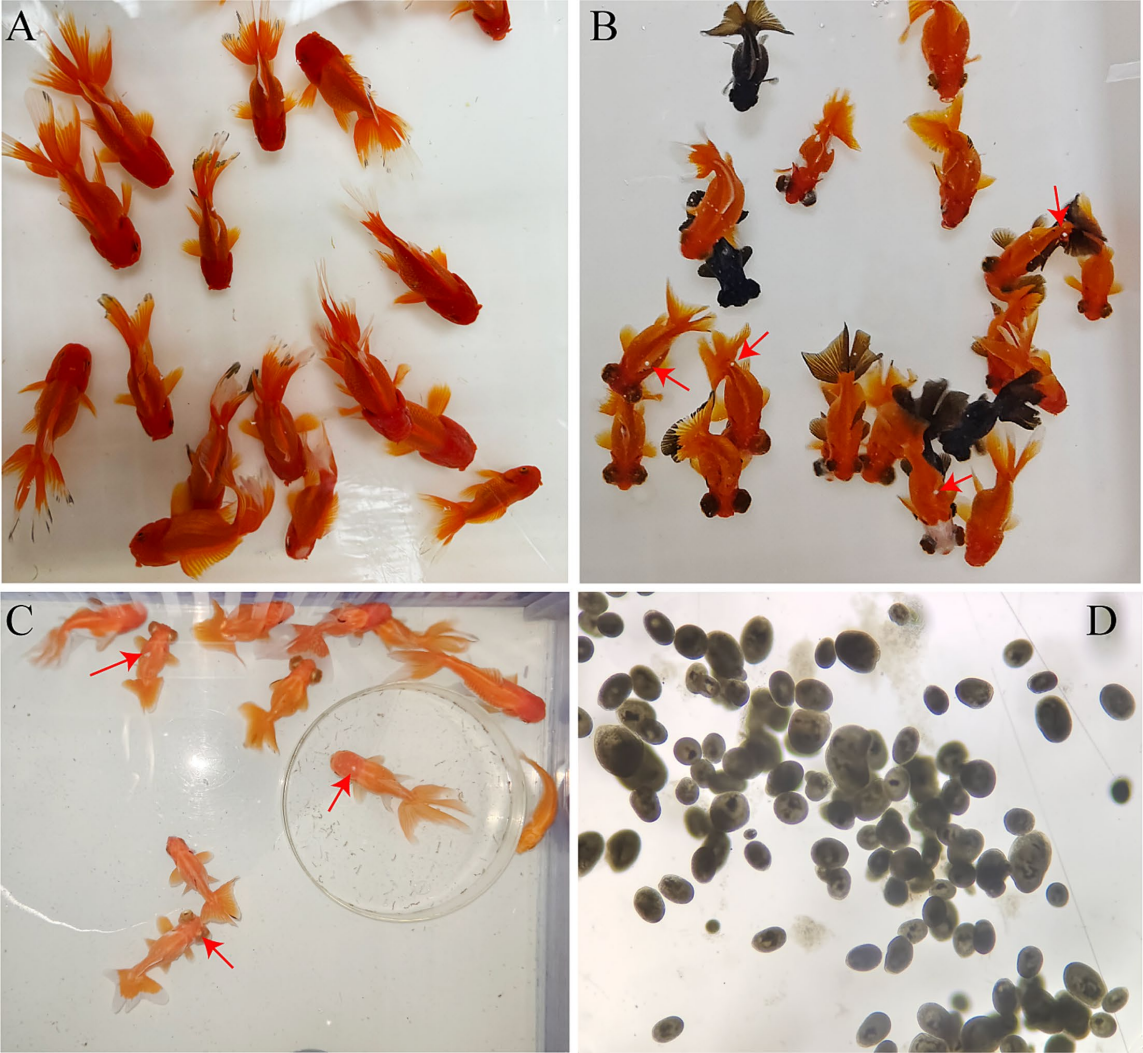
Infection status of the goldfish and morphology of *I. multifiliis.* **(A)** Goldfish in the control group. **(B)** Goldfish in the early stage of infection, red arrows to show white spots. **(C)** Behavior and body color of goldfish have changed after severe *I. multifiliis* infection, red arrows to show white spots. **(D)** Morphology of *I. multifiliis* during the peak infection period.

During the 14 days infection experiment, significant changes were observed in the behavior and body surface color of the goldfish. The body color of goldfish in the control group was very bright, and these goldfish swam in the same direction (Figure 1A). In the early stages of infection, the behavior of goldfish was normal, but some white spots on body surface were visible (Figure 1B). From the fifth day of infection, an outbreak of *I. multifiliis* occurred. The white spots were visible on the skin and fins of the goldfish (Figure 1C). The behavior of goldfish was becoming abnormal. Most fish often rubbed against the tank wall, swimming in different directions, and some showed unbalanced “backstroke.” Goldfish with severe infections developed white mucous membranes on their skin. Their respiratory rate was found to be significantly reduced by observing the movement of operculum. Then the goldfish was sinking to the bottom and floating with waves. The morphology of *I. multifiliis* was shown in Figure 1D.

Meanwhile, the average number of *I. multifiliis* per goldfish and the number of dead goldfish each day was recorded (Table 1). The number of *I. multifiliis* on goldfish reached its peak on the 7th day of infection, which was 1,280, and then gradually decreased. However, there were still 660 trophonts on the last day (14th day) of the infection experiment. The total mortality rate of goldfish was 22.22%.

**Table 1:** Statistics on the infection of *I. multifiliis* in goldfish.

Time (day)	The average number of *I. multifiliis* per goldfish	The number of dead goldfish
1	0	0
2	0	0
3	1	0
4	4	1
5	48	0
6	436	2
7	1,280	2
8	1,202	2
9	1,010	4
10	880	1
11	862	1
12	830	3
13	600	2
14	660	2

### 3.2 Histopathology of gill and intestine

**Figure 2:**
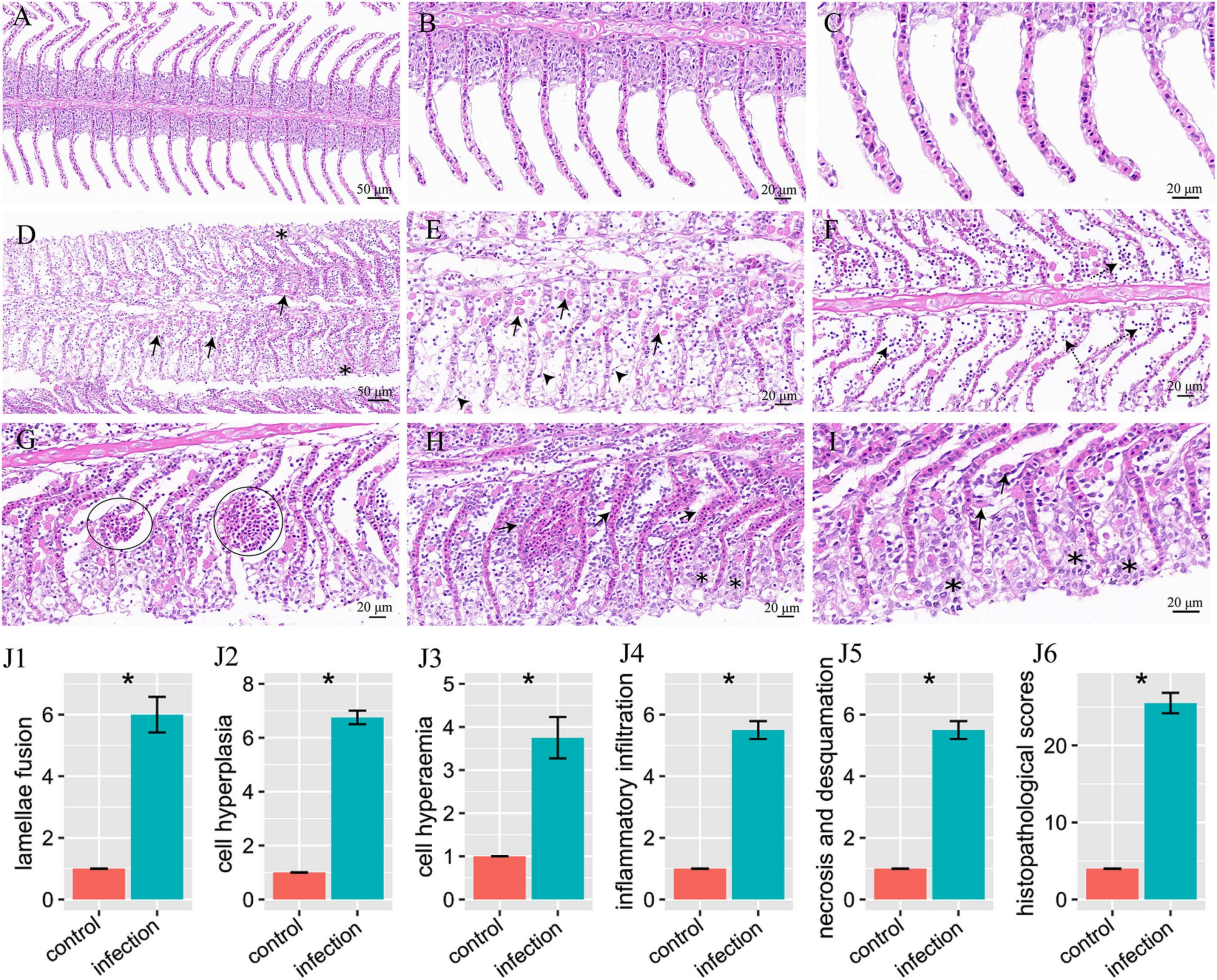
H&E staining results of goldfish gills and statistical results of gill histopathological score. **(A–C)** Gills of the control group, magnified 20×, 40×, 63×, respectively. **(D–I)** Gills of the infection group. **(D)** Eosinophilic granulocyte increases (arrows), cell hyperplasia and swollen gill filament (*). **(E)** Magnification of **(D)** to show eosinophilic granulocyte (arrows) and epithelial cell detachment (arrowheads). **(F)** To show inflammatory cell infiltration (dotted arrows). **(G)** Vascular dilation and aneurysmal lesions (circles). **(H)** Hyperemia (arrows) and swollen gill lamella (*). **(I)** To show cell necrosis, chromatin decrease, edge shift (*), and eosinophilic granulocyte (arrows). **(J1–J6)** Histopathological score of lamellae fusion, cell hyperplasia, cell hyperemia, inflammatory infiltration, necrosis and desquamation, and total histopathological scores, respectively.

According to the H&E staining results, after 14 days *I. multifiliis* infection experiment, the gills of the control and infection group showed significant histological characteristics. The structures of gill filaments and gill lamellae of the control group were clear and regularly arranged (Figure 2A). The morphology and structure of respiratory epithelium and red blood cells were also very complete (Figures 2B,C). However, compared with the control group, the gills of *I. multifiliis* infected group showed gill filament swelling, eosinophilic granulocyte increases and cell hyperplasia (Figure 2D). Upon magnification, the epithelial cell detachment in Figure 2D can be clearly observed (Figure 2E). Meanwhile, the gills of infected group also showed inflammatory cell infiltration (Figure 2F). Vascular dilation and aneurysmal lesions, along with hyperaemia and swollen gill lamella also can be seen (Figures 2G,H). The gills of *I. multifiliis* infected group also showed the sign of cell necrosis, chromatin decrease and chromatin edge shift (Figure 2I).

**Figure 3:**
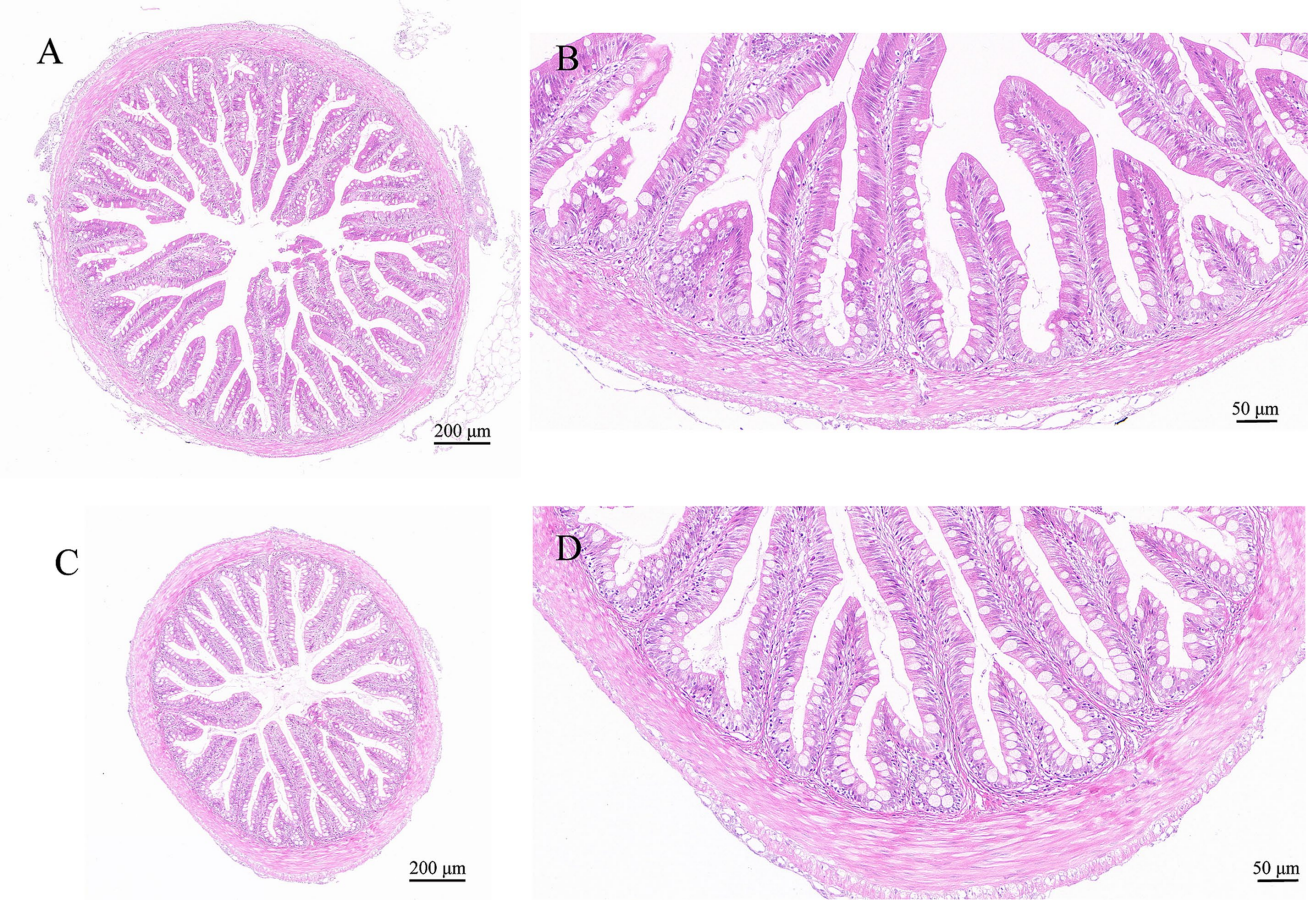
H&E staining results of goldfish intestines. **(A,B)** Intestine of the control group. **(C,D)** Intestine of the *I. multifiliis* infected group.

By histopathological scoring, the pathological changes including lamellae fusion, cell hyperplasia, cell hyperaemia, inflammatory infiltration, necrosis and desquamation between the control group and *I. multifiliis* infected group showed significant difference (Figures 2J1–J6). However, the H&E staining results of intestine between the control group and *I. multifiliis* infected group had no difference (Figure 3).

### 3.3 ASVs distribution

**Figure 4:**
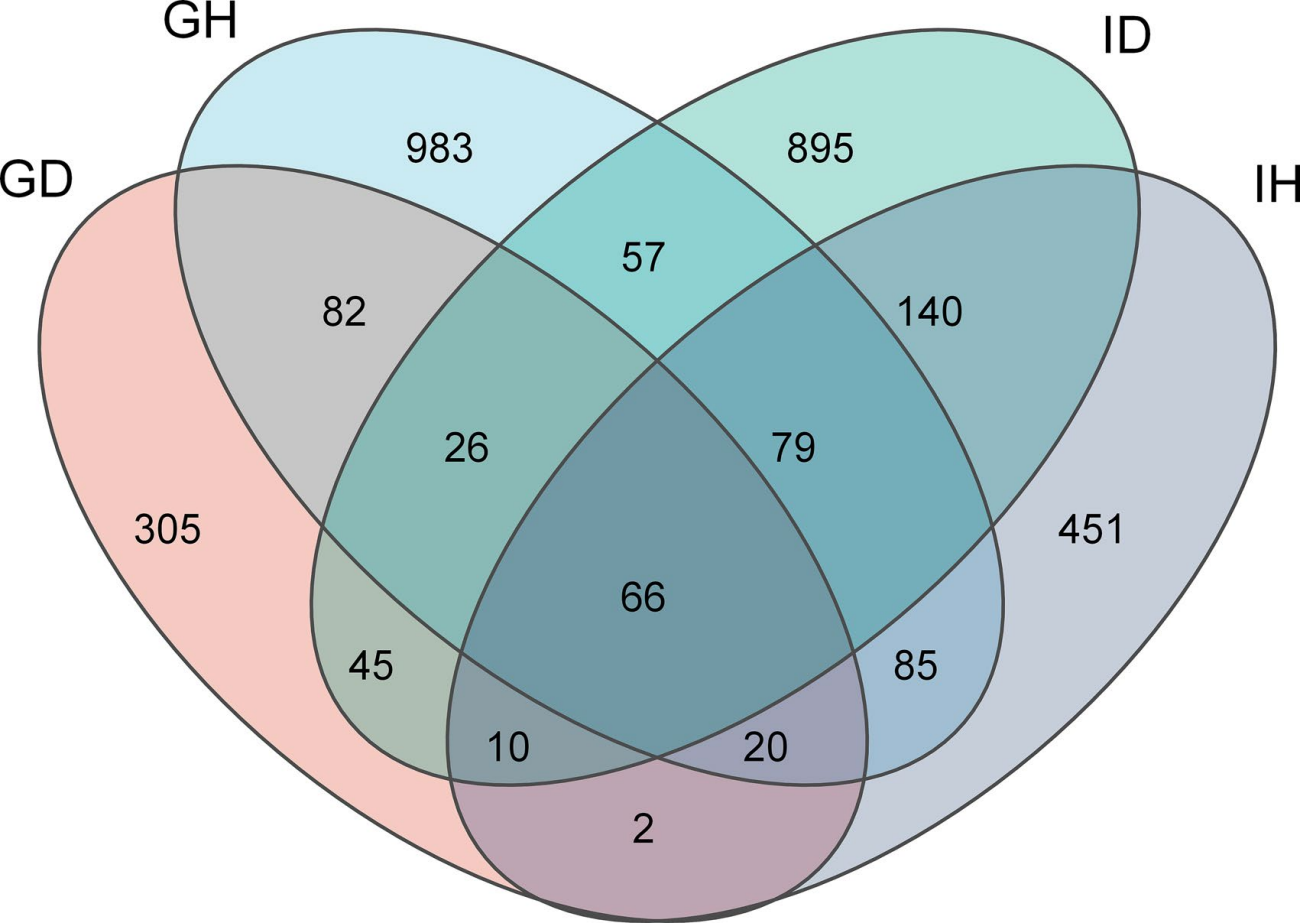
Venn diagram of gill and intestinal microbiota ASVs distribution in *I. multifiliis*-infected group and control group. GH: gills of the healthy/control group, GD: gills of the diseased/infected group; IH: intestines of the healthy/control group, ID: intestines of the diseased/infected group.

A total of 1,392,420 sequences were obtained after decontamination. Sequencing results showed that the average sequencing depth was 42,751. As seen in the Venn diagram (Figure 4), 3,636 ASVs were identified as either shared or unique among the four groups. The number of unique ASVs in the GH group (gills of the healthy/control group) was 983, while it was 305 in the GD group (gills of the diseased/infected group). The number of ASVs shared by GH group and GD group was 82. Additionally, the numbers of unique ASVs in the IH group (intestines of the healthy/control group) and in the ID group (intestines of the diseased/infected group) were 451 and 895, respectively. The number of ASVs shared by the two groups was 140. Moreover, there was 66 ASVs shared by the four groups. Overall, the numbers of ASVs in the GH, GD, IH, and ID groups were 1,398, 556, 853, and 1,318, respectively.

### 3.4 The impact of *Ichthyophthirius multifiliis* on the alpha diversity of gill and intestinal microbiota

**Figure 5:**
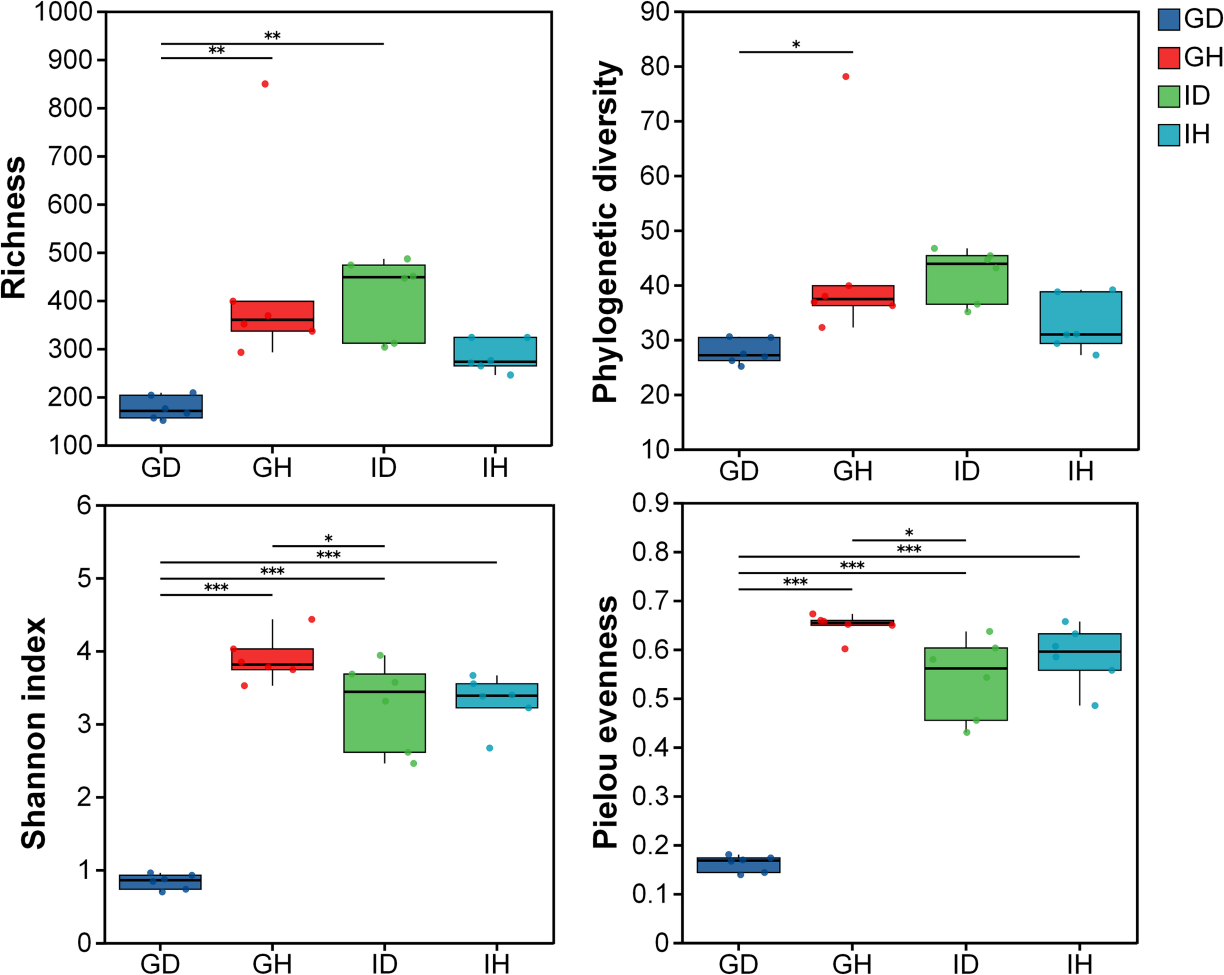
The alpha diversity of gill and intestinal microbiota in *I. multifiliis*-infected group and control group. * representing *p* < 0.05, ** representing *p* < 0.01, *** representing *p* < 0.001; GH: gills of the healthy/control group, GD: gills of the diseased/infected group; IH: intestine of the healthy/control group, ID: intestine of the diseased/infected group.

As for the alpha diversity, richness, phylogenetic diversity, shannon index, and pielou evenness indices were calculated and compared (Figure 5). The results showed that the alpha diversity in the GD group was significantly lower than that in the GH group (*p* < 0.05), whereas no significant differences were found between the IH and ID groups. Meanwhile, the richness, shannon, and pielou evenness indices in the GD group were significantly lower than that in the ID group. The four indices had no significant differences between the GH and IH groups.

### 3.5 The impact of *Ichthyophthirius multifiliis* on the beta diversity of gill and intestinal microbiota

**Figure 6:**
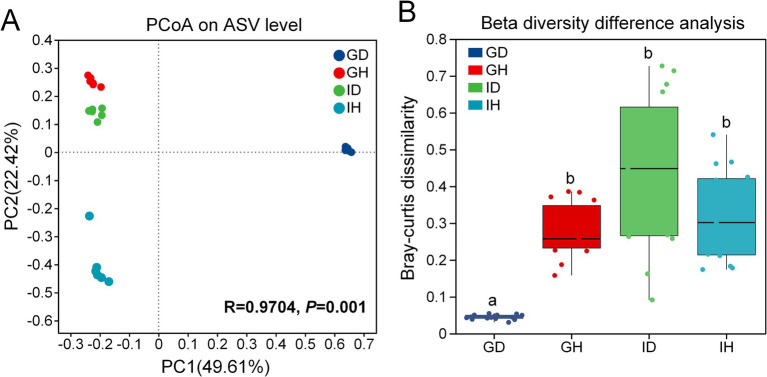
**(A)** the Principal Co-ordinates Analysis (PCoA) on ASV level. **(B)** Beta diversity difference analysis The beta diversity of gill and intestinal microbiota in *I. multifiliis*-infected group and control group. GH: gills of the healthy/control group, GD: gills of the diseased/infected group; IH: intestines of the healthy/control group, ID: intestines of the diseased/infected group.

The Principal Co-ordinates Analysis (PCoA) results revealed that samples from different groups were distributed in different regions (Figure 6A) (PC1 = 49.61%, PC2 = 22.42%). It indicated that the bacterial community composition of the four groups were significantly different (*p* = 0.001). As shown in the Figure 6B, beta diversity in the GD group was significantly lower than that in the other three groups including GH, ID and IH groups.

### 3.6 Microbiota composition in *Ichthyophthirius multifiliis*-infected and control group

**Figure 7:**
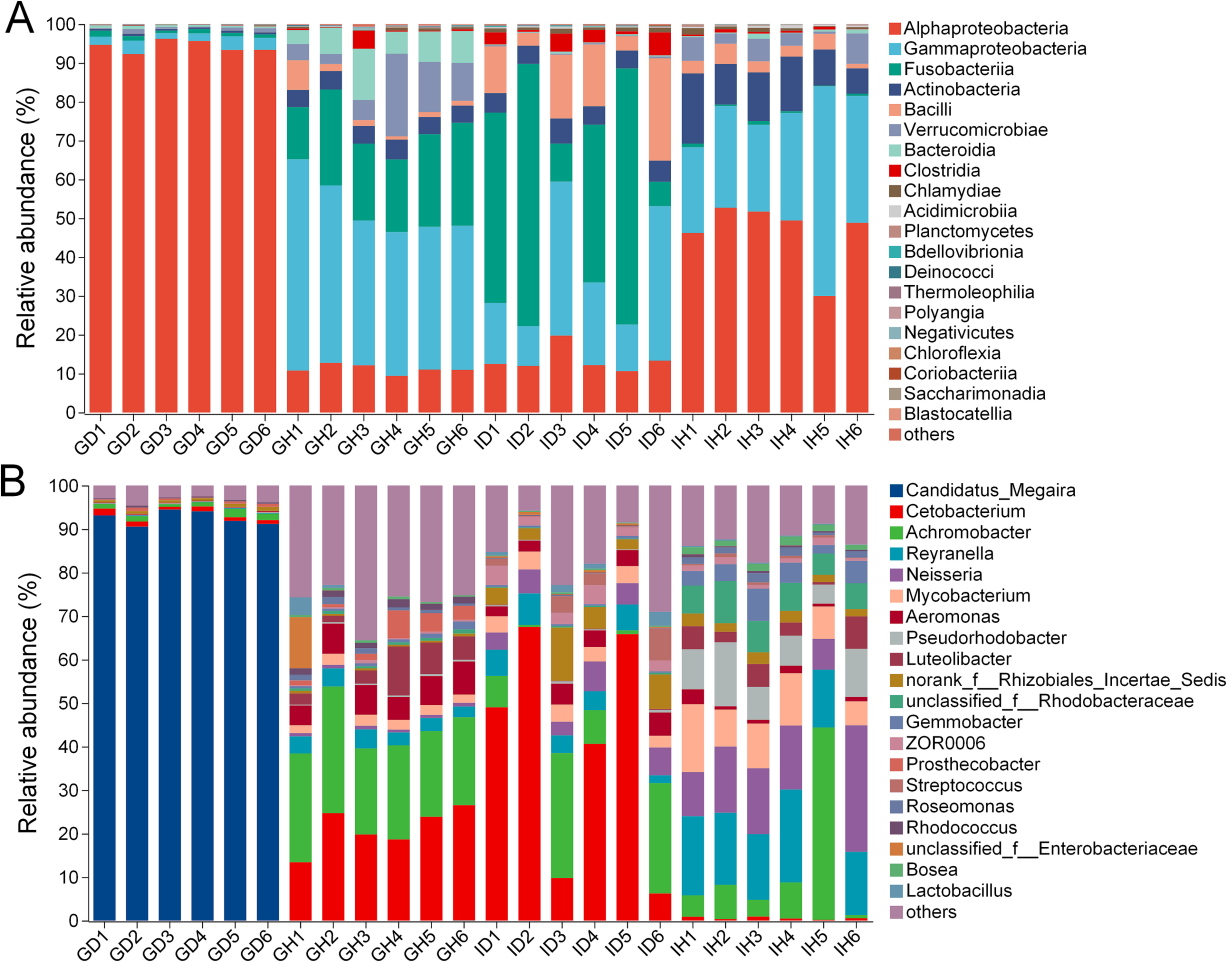
The relative abundance of gill and intestinal microbiota at class **(A)** and genus **(B)** levels of *I. multifiliis*-infected group and control group. GH: gills of the healthy/control group, GD: gills of the diseased/infected group; IH: intestine of the healthy/control group, ID: intestine of the diseased/infected group.

At the class level, Alphaproteobacteria (over 90%) was the most abundant bacteria in the GD group (gills of the diseased/infected group), while Gammaproteobacteria (41.40%), Fusobacteriia (21.17%), and Alphaproteobacteria (11.18%) were the relatively abundant taxa in the GH group (gills of the healthy/control group) (Figure 7A; Table 2). In the ID group (intestines of the diseased/infected group), Fusobacteriia, Gammaproteobacteria, Alphaproteobacteria, and Bacilli were the top four relatively abundant taxa, which accounted for 39.88%, 23.11%, 13.40%, and 12.96%, respectively. Alphaproteobacteria, Gammaproteobacteria, and Actinobacteria were the top three abundant taxa in the IH group (intestines of the healthy/control group), accounting for 46.49%, 30.84%, and 11.87%, respectively (Table 2).

**Table 2:** Bacteria abundant at the class level.

Group	GD	GH	IH	ID
Bacteria				
Alphaproteobacteria	>90%	11.18%	46.49%	13.40%
Gammaproteobacteria	–	41.40%	30.84%	23.11%
Fusobacteriia	–	21.17%	–	39.88%
Bacilli	–	–	–	12.96%
Actinobacteria	–	–	11.87%	–

^– representing non-dominant bacteria.^

At the genus level, *Candidatus Megaira* was the most abundant bacteria in the GD group, while *Cetobacterium* and *Achromobacter* were the relatively abundant taxa in the GH group (Figure 7B). *Cetobacterium* and *Achromobacter* also were the relatively abundant taxa in the ID group. *Reyranella*, *Neisseria*, and *Achromobacter* accounted for 16.51%, 15.22%, and 11.64%, respectively, which were the three most relatively abundant taxa in the IH group.

### 3.7 Species difference analysis

**Figure 8:**
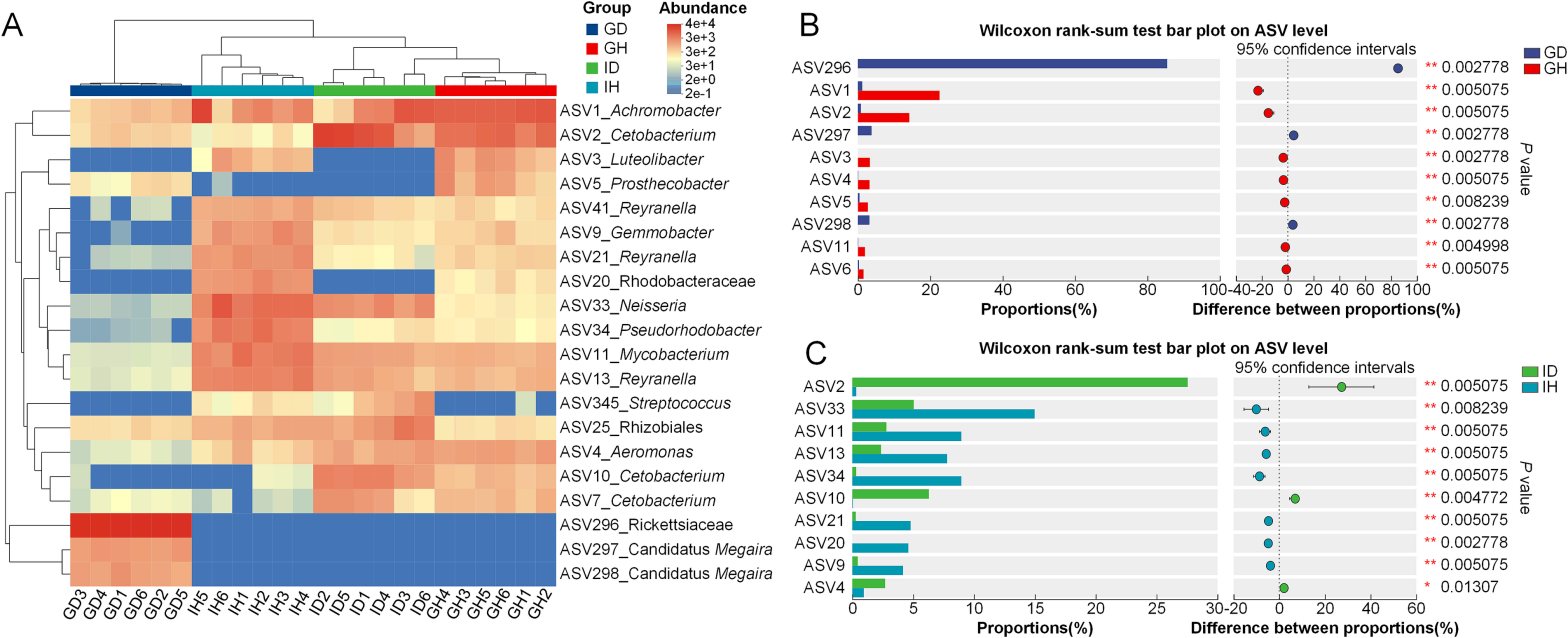
Community composition analysis of gill and intestinal microbiota in *I. multifiliis*-infected group and control group. **(A)** Heatmap showing the distribution of top dominant species in the four different groups. **(B,C)** Bar chart displaying the differences in average relative abundance of the same ASVs between groups.

Meanwhile, the distribution and difference of these top dominant species were shown in the Figure 8. It indicated that the difference of the four groups was significant. The content of ASV296_Rickettsiaceae in the GD group was significantly higher than that in the GH group. The relative amounts of ASV1_*Achromobacter* and ASV2_*Cetobacterium* in the GD group were lower than that in the GH group (Figure 8B). As for the intestinal microbiota, the relative abundance of ASV2_*Cetobacterium* and ASV4_ *Aeromonas* was significantly higher in the ID group than that in the IH group. Besides, the content of ASV33_*Neisseria*, ASV11_*Mycobacterium*, ASV13_*Reyranella*, ASV34_*Pseudorhodobacter*, ASV21_*Reyranella*, and ASV20_Rhodobacteraceae was significantly higher in the IH group, compared with the ID group (Figure 8C).

**Figure 9:**
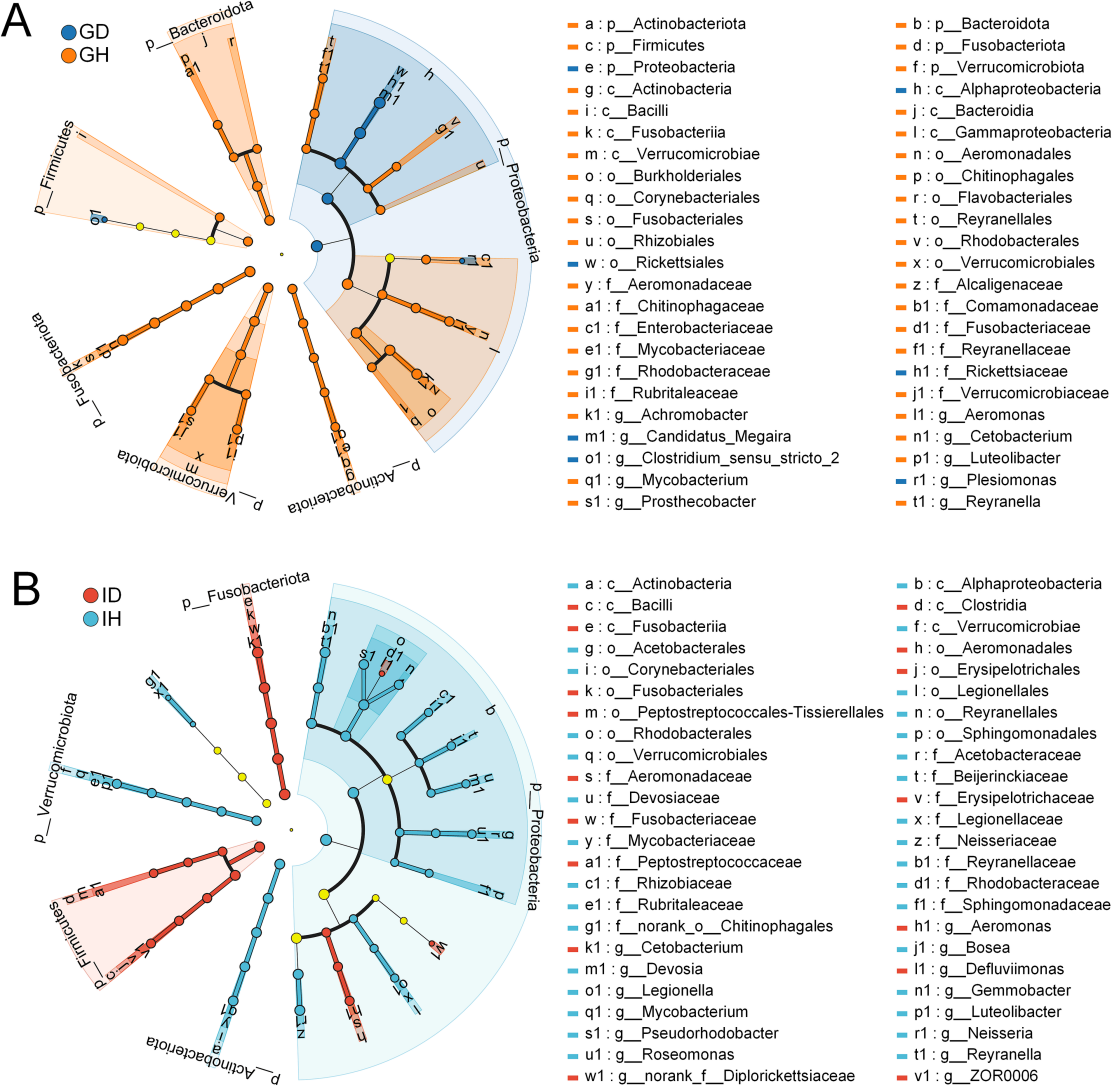
Linear discriminant analysis Effect Size (LEfSe) of gill and intestinal microbiota in *I. multifiliis*-infected group and control group. **(A)** Showing the difference between GD and GH group. **(B)** Showing the difference between ID and IH group. The light yellow nodes indicating no significant differences among different groups.

Additionally, LEfSe was used to analyze the differences at multiple levels (Figure 9). It showed that microflora of the taxa including the phylum Proteobacteria, class Alphaproteobacteria, order Rickettsiales, family Rickettsiaceae, genus *Candidatus Megaira*, *Clostridium sensu stricto 2*, and *Plesiomonas* were significantly enriched in the GD group (Figure 9A). Compared with the IH group, the microorganisms in the class Bacilli, Clostridia, and Fusobacteria showed significant enrichment in the ID group, which also had a notable impact on the intergroup differences (Figure 9B).

### 3.8 Functional prediction of microbiota in infected group and control group

**Figure 10:**
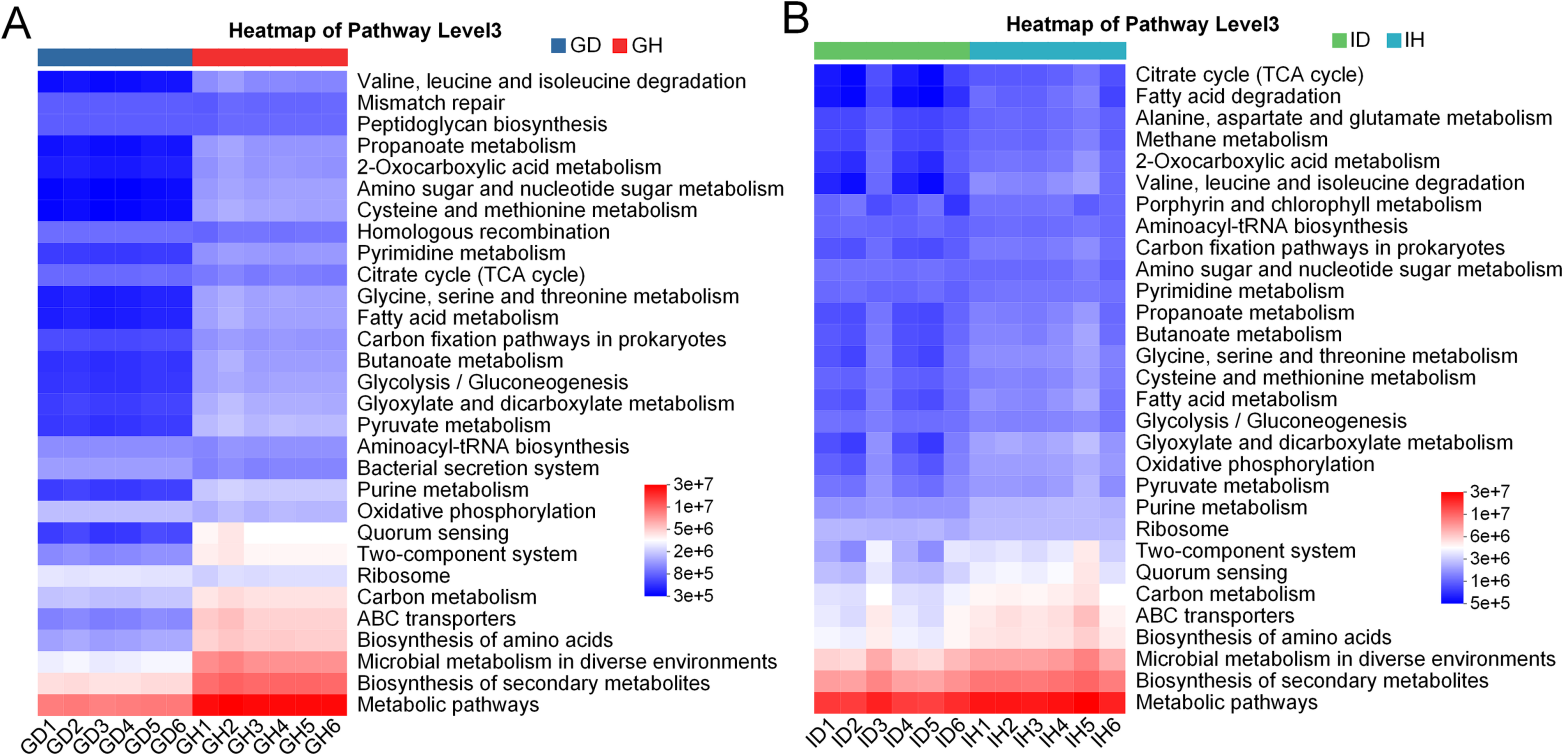
KEGG annotation of gill **(A)** and intestinal **(B)** microbiota in *I. multifiliis*-infected group and control group.

Based on the microbial community data, functional prediction was conducted to preliminarily explore the functions of microbial communities in the different groups (Figure 10). The KEGG result showed that there was a notable difference in the number of microorganisms related to main metabolism pathways including valine, leucine and isoleucine degradation, glycine, serine and threonine metabolism, citrate cycle (TCA cycle), fatty acid metabolism, pyruvate metabolism, oxidative phosphorylation, and carbon metabolism between the GD and GH groups (Figure 10A). These pathways have also been annotated in the ID and IH groups, however, the difference in these pathways between the ID and IH groups was not obvious (Figure 10B). Besides, both the ID and IH groups showed the function of methane metabolism.

## 4 Discussion

*Ichthyophthirius multifiliis* can infect a variety of common freshwater fish, and the “white spot disease” it causes, is considered as a fatal disease. In the present study, an artificial infection model using goldfish as the host of *I. multifiliis* was constructed, and the effects of *I. multifiliis* on host behavior, body color and histopathology, as well as the gill and gut microbiota, were further explored.

### 4.1 Histopathological changes in goldfish caused by *Ichthyophthirius multifiliis*

The histology characteristics of goldfish infected with and without *I. multifiliis* were analyzed. Compared with the uninfected goldfish, the gills of infected goldfish showed significantly lamellae fusion, cell hyperplasia, cell hyperaemia, inflammatory infiltration, necrosis and desquamation, which may directly induce the abnormal behavior of the *I. multifiliis* infected goldfish. Previous studies have reported the effects of histopathology of *I. multifiliis* on the wild snakehead murrel (*Channa striata*) (21), grass carp (*Ctenopharyngodon idella*) (22), largemouth bass (*Micropterus salmoides*) (23), channel catfish (*Ictalurus punctatus*) (24), rainbow trout (*Oncorhynchus mykiss*) (25), *Schizothorax macropogon* (26), white skirt tetra (*Gymnocorymbus ternetzi*) (27), as well as goldfish (*C. auratus*) (7). The damage to the primary lamellae and secondary lamellae caused by *I. multifiliis* is remarkably consistent across different species. Additionally, previous study also has indicated that goldfish infected by *I. multifiliis* exhibit an increase in the number of mucous cells in the gill tissue (7). Generally, fish gills play important roles in gaseous exchange, excretion, and circulation. The severe damage to the gills can directly lead to hypoxia in the host, eventually resulting in the death of the host.

In this study, *Ichthyophthirius multifiliis* infection led to an increase in eosinophilic granulocytes in the gills of goldfish. Eosinophilic granulocytes take important roles in host defense against parasitic protozoa and helminth (28, 29). Previous study on goldfish infected by another ectoparasite *Chilodonella hexasticha* showed that *C. hexasticha* infection also can increase the number of eosinophilic granulocytes, which may participate in resisting parasitic infections (30). Monogenean *Dactylogyrus lamellatus* not only affected the directly infected tissue gills, but also caused pathological changes in visceral tissues like liver, spleen, kidney, and intestines. It induced infiltration of a large number of immune cells into the mucosa and submucosa, leaded to a decrease in mucus-producing cells, and caused fracturing between the mucosa and the lamina propria (11). However, in this study, no pathological changes of the goldfish intestine were observed. The impact caused by *I. multifiliis* on intestine may manifest in other aspects.

### 4.2 The impact of *Ichthyophthirius multifiliis* on the gill and gut microbiota

Fish gills directly interact with aquatic environments, which contain a highly diverse group of microorganisms. When ectoparasites infect gills, the balance between fish and microbiota could be disrupted (31). The parasites and bacteria may have some interactions. In the previous studies, the relationship between *I. multifiliis* and *Edwardsiella ictaluri* was explored. It showed that *E. ictaluri* can survive and replicate inside the tomonts, resulting in high bacterial burdens in different organs and high mortality rates of channel catfish (32, 33). In this study, compared with the healthy/control group, the alpha and beta diversity of gill microbiota in *I. multifiliis* infected group was significantly lower. However, there was no significant difference observed in the alpha and beta diversity of the intestinal microbiota between the control group and *I. multifiliis* infected group. It could be due to the differences in parasitic sites. Previous studies found that internal parasites of fish or mammals that parasitize the gut can induce a decrease in gut microbiota diversity (12, 34–36). *Ichthyophthirius multifiliis* directly parasitize fish gills, thus having the opportunity to affect the diversity of gill microbiota. Meanwhile, from the venn diagram analyses, the ASVs results showed a decrease in gills of *I. multifiliis* infected goldfish (GH group), providing support for the significant decrease of alpha and beta diversity. These results suggest that *I. multifiliis* can directly affect the microbial diversity of the parasitic site gills.

As for the microbiota composition, in the gills of the healthy goldfish, the predominant bacteria at class level were Gammaproteobacteria, Fusobacteriia, and Alphaproteobacteria, respectively, which was consistent with previous reports (37, 38). However, in the gills of *I. multifiliis* infected goldfish, Alphaproteobacteria accounted for more than 90%, which showed an imbalance of the bacterial community. *Candidatus Megaira* was the most abundant genus within the class Alphaproteobacteria in the gills of *I. multifiliis* infected goldfish. *Cetobacterium* and *Achromobacter* were the predominant genera in the control goldfish, consistent with the previous study (39). Microbes from *C. Megaira* (Rickettsiales) are well-known as the endosymbionts of ciliates and other eukaryote, and the causative agents for some human diseases like typhus and Rocky Mountain spotted fever (40–42). It has been proved that *C. Megaira* is extensively distributed within *I. multifiliis*, being dispersed throughout the cytoplasm of trophonts and also present in majority theronts (43, 44). From these results above, it can be inferred that the significant increase of *C. Megaira* in goldfish gills may originate from *I. multifiliis* trophonts. Further research is needed to study the deep relationship between *C. Megaira* and *I. multifiliis*, providing new insights for the prevention and treatment of Ichthyophthiriasis.

Although no significant difference in intestinal microbiota diversity was detected between the *I. multifiliis* infected and control goldfish, it’s noteworthy that *I. multifiliis* infection corresponded to an increase in the abundance of several bacteria like *Cetobacterium* and *Aeromonas*. Meanwhile, we found that *Cetobacterium* and *Achromobacter* were the relatively abundant genera in the intestinal of *I. multifiliis* infected goldfish. Previous studies showed that *Cetobacterium* is the predominant genus within the gastrointestinal microbiota of goldfish and other freshwater fish, exhibiting a significant role in cellulose degradation, metabolic homeostasis, and is beneficial for the host (45–47). *Aeromonas* species primarily pose a threat to poikilothermic creatures, with mesophilic strains increasingly recognized as significant pathogens in humans, causing extraintestinal and systemic infections (48). Among the *Aeromonas* species, *Aeromonas hydrophila*, identified as an opportunistic pathogen, could induce intestinal inflammation in a variety of farmed fish including grass carp (*Ctenopharyngodon idella*) and rainbow trout (*Oncorhynchus mykiss*) (49, 50). *Achromobacter* is a type of opportunistic pathogen and potentially impacted fish health status (51, 52). Thus, it can be inferred that the increased relative abundance of *Aeromonas* and *Achromobacter* may threaten the health of host goldfish. In other words, *I. multifiliis* infection may increase the risk of dysbiosis of intestinal flora and enteritis.

## 5 Conclusion

In summary, we found that *I. multifiliis* infection could induce changes in the gill histopathological characteristics, gill and gut microbiota. The abnormal behavior of host may be attributed to the alteration of gill histopathology. Meanwhile the increase of genus *Candidatus Megaira* in gill microbiota was associated with the severe infection of *I. multifiliis*. The increased relative abundance of *Aeromonas* and *Achromobacter* in the intestine of *I. multifiliis* infected goldfish may be a threaten to goldfish health status. This study lay a foundation for further research on the interaction between *I. multifiliis* and host microbiota, which will promote the prevention and control of Ichthyophthiriasis.

## Data Availability

The sequencing data of this study has been uploaded to NCBI (https://www.ncbi.nlm.nih.gov/) under the BioProject ID: PRJNA1193790.
